# Brain computer interface training with motor imagery and functional electrical stimulation for patients with severe upper limb paresis after stroke: a randomized controlled pilot trial

**DOI:** 10.1186/s12984-024-01304-1

**Published:** 2024-01-20

**Authors:** Iris Brunner, Camilla Biering Lundquist, Asger Roer Pedersen, Erika G. Spaich, Strahinja Dosen, Andrej Savic

**Affiliations:** 1 Department of Clinical Medicine, Hammel Neurocenter and University Hospital, Aarhus University, Voldbyvej 12, 8450 Hammel, Denmark; 2 Hammel Neurocenter and University Research Hospital, 8450 Hammel, Denmark; 3 University Research Clinic for Innovative Patient Pathways, Diagnostic Centre, Silkeborg Regional Hospital, 8600 Silkeborg, Denmark; 4 Department of Health Science and Technology, Aalborg University, 9220 Aalborg, Denmark; 5 Science and Research Centre, University of Belgrade-School of Electrical Engineering, Belgrade, 11000 Serbia

## Abstract

**Background:**

Restorative Brain–Computer Interfaces (BCI) that combine motor imagery with visual feedback and functional electrical stimulation (FES) may offer much-needed treatment alternatives for patients with severely impaired upper limb (UL) function after a stroke.

**Objectives:**

This study aimed to examine if BCI-based training, combining motor imagery with FES targeting finger/wrist extensors, is more effective in improving severely impaired UL motor function than conventional therapy in the subacute phase after stroke, and if patients with preserved cortical-spinal tract (CST) integrity benefit more from BCI training.

**Methods:**

Forty patients with severe UL paresis (< 13 on Action Research Arm Test (ARAT) were randomized to either a 12-session BCI training as part of their rehabilitation or conventional UL rehabilitation. BCI sessions were conducted 3–4 times weekly for 3–4 weeks. At baseline, Transcranial Magnetic Stimulation (TMS) was performed to examine CST integrity. The main endpoint was the ARAT at 3 months post-stroke. A binominal logistic regression was conducted to examine the effect of treatment group and CST integrity on achieving meaningful improvement. In the BCI group, electroencephalographic (EEG) data were analyzed to investigate changes in event-related desynchronization (ERD) during the course of therapy.

**Results:**

Data from 35 patients (15 in the BCI group and 20 in the control group) were analyzed at 3-month follow-up. Few patients (10/35) improved above the minimally clinically important difference of 6 points on ARAT, 5/15 in the BCI group, 5/20 in control. An independent-samples Mann–Whitney U test revealed no differences between the two groups, p = 0.382. In the logistic regression only CST integrity was a significant predictor for improving UL motor function, p = 0.007. The EEG analysis showed significant changes in ERD of the affected hemisphere and its lateralization only during unaffected UL motor imagery at the end of the therapy.

**Conclusion:**

This is the first RCT examining BCI training in the subacute phase where only patients with severe UL paresis were included. Though more patients in the BCI group improved relative to the group size, the difference between the groups was not significant. In the present study, preserved CTS integrity was much more vital for UL improvement than which type of intervention the patients received. Larger studies including only patients with some preserved CST integrity should be attempted.

## Background

Around 50% of patients with stroke suffer from limited dexterity in the affected upper limb (UL), ranging from slightly reduced dexterity to paralysis [1, 2]. Almost half of the patients with initial severe paresis, without a functional hand, and paralysis do not reach a satisfactory level of UL motor function, leaving many with an unusable UL and dependent on help [3]. Since most daily life activities are bimanual, impaired UL function compromises daily activities, quality of life, and vocational reintegration. Intensive training during the first weeks to months is crucial for regaining motor function [4]. During this period, the brain is most susceptible to reorganization [5]. However, it can be challenging to provide meaningful training for patients who can hardly move their UL voluntarily. Contrary to patients with some UL function who can participate in interventions such as constraint-induced movement therapy or task-specific training, there is a paucity of evidence-based and effective interventions for patients with severe paresis. Treatment options with some benefit include mirror therapy, mental practice, functional electrical stimulation, and advanced technologies, such as EMG biofeedback, robotic assistance, and exoskeletons, usually only available in specialized rehabilitation centers [6–8]. A relatively recent treatment alternative are restorative brain–computer interface (BCI) systems based on new treatment strategies that pair brain activity with contingent sensory feedback. Feedback modalities may include visual, motor and somatosensory stimulation. Visual feedback can involve 3D computer graphics (e.g., virtual arm model movement on the screen) or real object manipulations, while motor and somatosensory stimulation can be delivered by functional electrical stimulation (FES) or robotic (e.g. exoskeleton) devices executing/assisting the desired movements [9]. FES stimulates paretic sensorimotor nerves and induces artificially generated movements and afferent sensory feedback. Thereby, such restorative BCIs create a real-time feedback loop that can facilitate plasticity by strengthening neural connections [10–12]. Moreover, principles of experience-dependent neural plasticity are applied since patients are cognitively engaged in the training comprising many challenging repetitions, which is particularly relevant for patients with severe paresis who are unable to participate in more active interventions [13].

Several reviews conclude that the evidence for BCI training is still scarce, though promising, both as a restorative and an assistive approach [14–17]. Different systems and heterogeneous patient groups, mostly in the chronic phase after stroke, make comparisons challenging. Still, improvements in UL motor function in patients in the chronic phase were significant and seemed to last after completing BCI-based therapy [18–20], though it can be debated if these improvements were clinically meaningful. In a recent study, BCI training combined with FES was found to increase resting state connectivity between the hemispheres and within the motor network, which correlated with improvements in UL motor function in chronic patients [21]. Other studies have found a stronger desynchronization of alpha and beta bands in the ipsilesional hemisphere after BCI training [22, 23], and emphasized the importance of using BCI methods to improve the sensorimotor rhythms associated with movement preparation, as an alternative to modifying the brain activity during movement [24].

However, the evidence of therapeutic effects from randomized controlled clinical trials including BCI technology for different patient populations is still insufficient [16]. Few small-scale studies targeted patients with severe paresis in the first weeks after stroke. Pichiorri et al. [22] included 28 patients and found larger improvements for the BCI group in combination with visual feedback than for the control group. Morrone et al. [25] included eight severely impaired individuals in the subacute stage but focused on usability only.

Demonstrating possible treatment effects in the subacute phase can be challenging due to spontaneous biological recovery [26]. Furthermore, patients in the subacute phase frequently suffer from multiple impairments and psychological distress, making it more challenging to achieve levels of training intensity even remotely close to those suggested by pre-clinical research [27]. Most importantly, not all patients with severe UL paresis may have the underlying biological capacity in terms of cortico-spinal tract integrity to respond to the BCI intervention [28]. In this context, BCI training seems promising, but the evidence is insufficient and challenging to generate.

In this study, we examined the effectiveness of BCI training for patients with severe upper limb paresis in the subacute phase after stroke. Moreover, we investigated potential changes in cortical activation in the BCI group. We hypothesized that BCI training combined with visual feedback and FES would result in better UL recovery in the BCI group. We also expected changes in motor imagery-related electroencephalography (EEG) patterns towards enhanced laterality of the event-related sensorimotor oscillations when approaching the end of therapy in the BCI group, coinciding with the UL recovery process. Furthermore, we hypothesized better UL recovery for patients with preserved cortico-spinal tract (CST) integrity, regardless of the type of intervention.

## Methods

### Design and setting

This study was a randomized controlled pilot trial. Forty patients admitted to rehabilitation at a specialized neurorehabilitation hospital were randomly allocated to either intervention with BCI training or standard control treatment. Patients were assigned with the help of a computerized randomization system with varying block sizes provided by a data-managing web application (REDcap™). All assessments were conducted by therapists blinded to the group allocation. It was not possible to blind patients. The study was approved by the Regional Ethics Committee for the Central Jutland Region in Denmark (registration number 1-16-02-173-19). All participants provided informed written consent. The study was prospectively registered at Clinicaltrials.gov NCT04071587.

### Eligibility criteria

Inclusion criteria: Adults with first-ever or former stroke as confirmed by CT and/or MRI, without UL motor residuals, within 60 days after stroke onset, severe paresis or paralysis defined as < 13 on the Action Research arm Test (ARAT) [29], able to give informed consent, able to comply with the treatment protocol, premorbid modified Rankin scale ≤ 2 (self-reported).

Exclusion criteria*:* Other conditions limiting functional use of the affected UL, psychiatric/behavioral conditions that interfere with compliance to the protocol.

### BCI system

The BCI system used was RecoveriX (g.tec, Austria). It consisted of an EEG amplifier, a patient screen, a therapist computer, and two FES devices, one for the left and one for the right hand. The EEG system included a cap with 16 active electrodes (g.Nautilus PRO, g.tec, Austria) set up according to the international 10/20 system. EEG channel locations were FC5, FC1, FCz, FC2, FC6, C5, C3, C1, Cz, C2, C4, C6, CP5, CP1, CP2, and CP6 while the reference was placed on the right earlobe and the ground at FPz.

### The BCI intervention

The BCI intervention was provided as part of the usual UL rehabilitation and not as an add-on to standard training. The targeted number of training sessions in the intervention group was 12. The sessions were conducted 3–4 times a week over 3–4 weeks. The intervention was as follows: (1) The patient wearing an EEG cap was seated comfortably with both hands on a Table 2. (2) The electrodes for FES were mounted on both forearms to activate the left and right extensor digitorum communis muscles. (3) In front of the patient there was a computer screen where virtual hands were displayed. Before the training, the therapist instructed the patient to imagine opening (extension of the fingers and dorsal extension of the wrist) in either the left or right hand as randomly announced by the system. Actual hand movements should not be attempted according to the instruction manual of the system. In the RecoveriX training, one single movement lasted for 8 s, starting with an attention sound, followed by the instruction to imagine opening either the left or right hand after 2 s. The subsequent feedback phase started 1.5 s after the command and lasted for 4.5 s until the command “relax” is announced auditorily. Feedback was provided by the avatar hands on the screen and FES inducing the imagined UL movement. Each run consisted of 80 imagined movements. The first run was used to calibrate the system to patients’ brain waves (training of the EEG movement detector) and FES was always provided to the left or right UL according to the auditory cue. If patients could imagine the movement consistently enough a classifier was created and the training proceeded into the next phase. If not, the calibration had to be repeated. During the second and subsequent runs, FES-induced movement of the hand was only provided if left and right motor imagination could be discriminated by the system, i.e., the classification confidence reached a level of more than 50% (chance level).

**Fig. 1:**
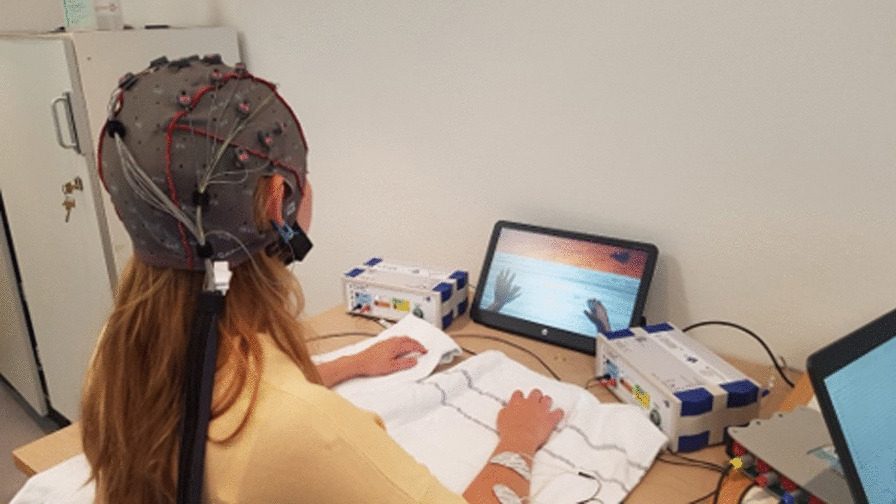
The RecoveriX BCI system

The FES was set to a frequency of 50 Hz and a pulse width of 300 µs and the stimulation intensity was individually adjusted to produce a visible movement, if possible, close to a full extension without causing discomfort (Fig. 1).

### Control treatment

Patients in the control group received standard physiotherapy and occupational therapy according to clinical guidelines during the same period. For patients with severe UL paresis the methods of choice comprised mirror therapy, passive movements, electrical and sensory stimulation.

### TMS examination

Patients in both groups underwent a TMS examination at baseline to assess the integrity of the corticospinal tract. Patients with contraindications to TMS were excluded from this examination. The TMS examination was conducted according to international guidelines [30]. Patients were seated with their ULs in a relaxed position. A MagStim 200 unit (Magstim Co. Ltd) delivered monophasic pulse waveforms using a 70-mm figure-of-eight coil. The target area was the ipsilesional primary motor area and the coil was directed to induce a posterior-to-anterior current flow. The stimulation intensity started at 50% of the maximal stimulator output (MSO) and was increased in 10% steps. The examiner moved the coil in approximately 1 cm steps (anterior, posterior, medial, lateral) to find the optimal location, and each area was stimulated 3–5 times with a given intensity. Electromyographic activity was recorded from the first dorsal interosseous and the extensor carpi radialis muscle of the affected UL using standard surface electrodes. The stimulus intensity was increased until motor-evoked potentials (MEP) could be consistently observed in one or both target muscles, or until 100% MSO was reached. If no MEPs were present at 100% MSO, the patients were asked to clench both their fists (or attempt to do so in case of the affected UL) to facilitate MEPs. Patients were classified as MEP+ if it was possible to elicit either passive or active MEPs with a peak-to-peak amplitude ≥ 50 µV at consistent latency in response to at least 5 consecutive stimuli [30, 31]. If this was not possible, patients were classified as MEP−. The examiner was blinded to the allocation of the patients.

### EEG data processing and analysis

For patients in the BCI group, EEG data from the BCI system calibration sessions were analyzed. Channels C3 and C4 were used for further analysis. Offline EEG processing was performed in MATLAB (R2020a, The MathWorks Inc., Natick, USA). Raw EEG was filtered (4th order Butterworth band-pass filter) to extract 3 frequency bands: alpha (8–13 Hz), beta (13–30 Hz), and whole alpha–beta range (8–30 Hz). The filtered EEG was squared and segmented with epochs ranging from 3 s before the cue to 4 s after the cue i.e. [− 3, 4] s, where 0 marks the cue instructing the patient to imagine opening either the left or right hand. Noisy trials were identified as the ones exceeding ± 150 µV threshold in band-pass filtered signals in the 0.1–30 Hz range. Additional manual inspection of band-power time courses of signals filtered in the 8–30 Hz range was implemented to remove noisy epochs.

Band-power time courses for each frequency band were averaged over each session and movement type (left/right). Event-related desynchronization/synchronization (ERD/ERS) values were calculated for each session according to the following equation [32]:

1 $$ERD/ERS\;(\%) = 100*\frac{Pmov-Pref}{Pref}$$

Pmov was calculated as the median value of the averaged band-power time course for each session and movement type, in the time interval [1, 4] s. Pref was calculated as the median value of the averaged band-power time course for each session and movement type, in the interval [− 3, − 1] s. By definition, ERD is a negative value representing the percent decrease of band-power during motor imagery compared to the resting state, and stronger (lower negative values) ERD reflects higher cortical activations during the MI tasks [32]. For the patients with right body side hemiparesis, ERD/ERS values were flipped for channels C3 and C4 as well as the markers for movement types, simulating that all the patients had left side hemispheres to simplify the presentation and interpretation of the results.

A laterality index (LI) was calculated for each patient, run, movement type, and frequency band, based on the following equation:

2 $$LI = \frac{ERDi-ERDc}{abs(ERDi)+abs(ERDc)}$$

ERDi denotes ipsilesional ERD while ERDc contralesional ERD. In this context, since the data were transformed to simulate left body side paresis in all patients, (i.e. right hemisphere injury), ERDi values were the ones extracted from C4 while ERDc values were extracted from C3 channel. By using Eq. 2, the LI approached a value of − 1 when the brain activity was either purely ipsilesional or 1 for purely contralesional activation.

Since the BCI therapy was conducted over multiple sessions, all extracted EEG parameters were averaged over the first fourth of the sessions and the last fourth of the sessions for each patient in order to obtain a single value for each parameter that represents the beginning and end of the therapy process. Taking into account the variability that may impact the results of a single session, we used the average of multiple sessions to represent a time point in the therapy process.

### Outcome measures

At baseline, medical and demographic information was collected, see Table 1. Assessments with clinical scales were performed at baseline and 3 months post-stroke. The main outcome measure was the Action Research Arm Test (ARAT) at three months post-stroke. The ARAT is widely applied and has excellent psychometric properties [33]. The ARAT is used to evaluate different aspects of UL motor function from gross to fine motor skills on a scale of 0–57 (best). A secondary outcome measure was the Fugl-Meyer Motor Assessment UL (FMA-UL) a measurement of UL impairment on a scale of 0–66 (best). [34]

**Table 1:** Demographic and clinical characteristics

	All (n = 40)	BCI (n = 19)	Control (n = 21)
Age, years, (SD)	56 (13)	56 (14)	57 (11)
Gender: male	25 (62%)	11 (44%)	14 (56%)
Days post stroke (SD)	33 (14)	31(10)	34 (16)
Stroke type: infarction/hemorrhage/both	13/25/2	8/11/0	5/14/2
Stroke location			
Cortical	13	6	7
Cortical and subcortical	5	2	3
Subcortical	20	10	10
Brainstem	2	1	1
Stroke severity			
NIHSS (SD)	15.9 (4.9)	15.1 (2.7)	15.9 (4.9)
Scandinavian Stroke Scale (SD)	24.0 (8.6)	25 (6)	23 (11)
Hemiparetic side, left	25 (62.5%)	11 (57.9%)	14 (66.7%)
Dominant side affected	14 (35%)	7 (36.8%)	7 (33%)
Able to move hand at onset	0	0	0
Able to walk at onset	0	0	0
Modified Rankin scale at baseline	4 (4/4)	4 (4/4)	4 (4/4)
ARAT score at baseline median (IQR)	0 (0)	0 (0)	0 (0)
FMA score at baseline median(Q1–Q3)	4 (2/4)	4 (2/4)	4 (2/4)
FIM score at baseline median (Q1–Q3)	47 (38/57)	43 (38/51)	47 (36/66)
Neglect present at enrolment, n (%)	8 (21%)	5 (27.8%)	3 (15.0%)
MEP+/MEP− (n = 32)	18/14	8/6	6/12

For the BCI group, EEG measures were: (1) ERD values for three frequency bands (8–30 Hz, 8–13 Hz, and 13–30 Hz), two imagery tasks (affected and less affected hand movement imagery), and two EEG channels, and (2) associated LI values for three frequency bands and for affected and less affected hand movement imagery.

### Data analysis

SPSS28 was used to analyze data from the clinical scales. Descriptive statistics were applied to describe demographic and baseline characteristics. Differences in ARAT and FMA scores within groups were explored using related samples Wilcoxon signed-rank test. Differences in improvement between the groups at three months post-stroke were compared with independent samples Mann–Whitney U tests. A per protocol analysis was conducted.

A binominal logistic regression was performed to assess the effect of treatment and CST integrity (MEP+/−) on the likelihood that patients will experience a clinically meaningful difference in UL function. The minimally clinically important difference (MCID) was set to 6 for the ARAT [35]. The significance threshold was set to 0.05. Since this was a pilot study, no power calculation had been performed.

The normality of ERD and LI data was tested using the Kolmogorov–Smirnov test. Paired samples t-test was employed for ERD analysis, while the Wilcoxon signed-rank test was used for LI analysis, to assess statistically significant differences between the beginning and the end of therapy.

## Results

**Fig. 2:**
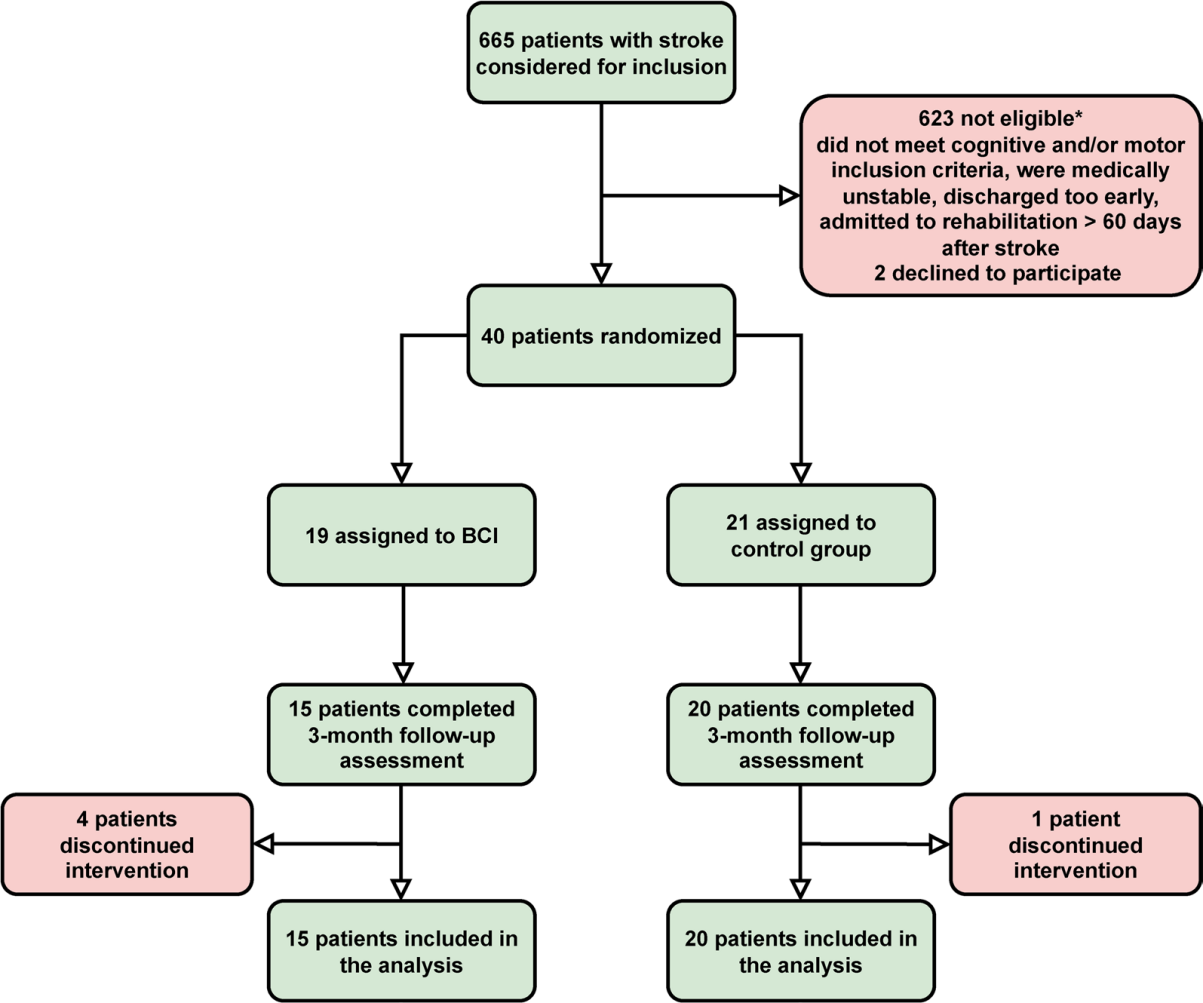
The flow of patients through the study

Forty patients were included from August 2019 to February 2022. Due to the Covid-19 pandemic, the inclusion had to be interrupted for approximately 6 months in total. Follow-up assessments could be obtained for 35 patients, 5 patients had discontinued their participation, 4 in the BCI group, and 1 in the control group, Fig. 2. Reasons for discontinuing were mainly unrelated to the study, such as worsening medical instability (n = 1), and early discharge (n = 2). One participant in the BCI group experienced muscle soreness due to the FES, which could not be resolved by decreasing the stimulation intensity. In the control group, one patient retracted their consent.

Patients in the two groups did not differ with regard to age, days post-stroke, stroke severity, and other characteristics at baseline. The ability to move the hand and to walk at stroke onset was based on a score of 0 for the hand item, and a score of 0 or 3 for the gait item on the Scandinavian Stroke Scale conducted at the acute hospital [36].

TMS examinations could be performed with 32 patients, seven had contraindications, and one declined. Demographic and medical information is presented in Table 1.

Patients in the BCI group received a mean of 10.6 (± 2.5) treatment sessions with the BCI system. As the BCI training was not an add-on but a part of UL training, the amount of training received was balanced. Patients received on average 11.15 h of physiotherapy and occupational therapy per week in both groups. The therapy did also address other impairments. The 3–4 h BCI training per week was part of the regular physiotherapy and occupational therapy sessions. A BCI session, including donning on doffing, lasted for 60 min and most patients did 160 repetitions during a session.

There was a statistically significant improvement within the groups from baseline to 3-month assessment, p = 0.012 for the control group, p = 0.007 for the BCI group for the main outcome measure ARAT. The median score for the BCI group changed from a baseline median (IQR) of 0 (0) for ARAT and 4 (3) for FMA to a 3-month follow-up score of 3 (11) for ARAT and 6.5 (21) for FMA. The control group had a baseline median (IQR) score of 0 (0) on ARAT and 4 (2) on FMA, and a 3-month follow-up score 0 (9) on ARAT and 4 (21) on FMA. The between-group differences were not statistically significant, neither for ARAT (p = 0.328) nor for FMA (p = 0.406).

**Fig. 3:**
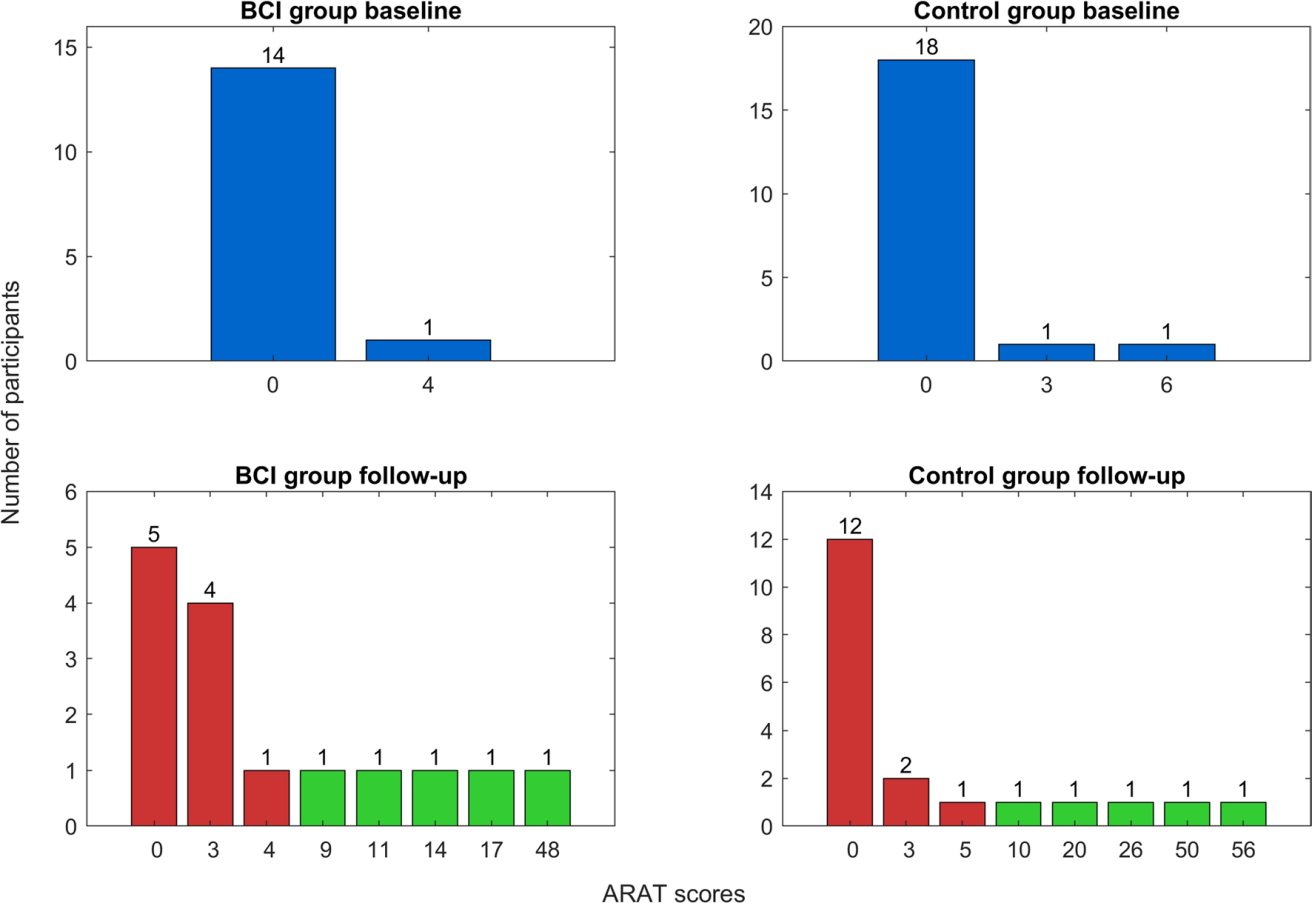
Distribution of scores on Action Research Arm Test (ARAT) at baseline (blue) and 3 months post-stroke for the BCI group and the control group. The green bars denote patients with improvement of at least 6 points on ARAT, the red bars with a change of less than 6 on ARAT

In general, only a few patients (10/35) in our sample improved and reached the MCID of 6 points change for ARAT at three months post-stroke. Of those, more patients were in the BCI group (5/15 = 33%, CI95% 12–62%) than in the control group (5/20 = 25%, CI95%: 9–49%). Figure 3 displays the numbers for the BCI and the control group.

The logistic regression with “allocation” (BCI/control) and “MEP status” (MEP+/MEP−) as independent variables, and a MCID of ≥ 6 on ARAT as dependent variable, was performed on 29 patients with complete data for both ARAT at three months and MEP status. An interaction term of allocation and MEP could not be estimated due to the limited sample size. The model explained 46.2% (Nagelkerke R^2^) of the variance in achieving an MCID on ARAT. Of the two independent variables, only MEP status was statistically significant. Patients with MEP+ had 29.4 higher odds to achieve some improvement in UL function, see Table 2.

**Table 2:** Logistic regression with allocation and MEP status as independent variables

	B	S.E	Wald	df	p-value	Exp (B)	95% CI for Exp (B)	
							Lower	Upper
Allocation (BCI)	− 0.668	1.066	0.393	1	0.531	0.513	0.063	4.140
MEP status (+)	3.381	1.252	7.293	1	0.007	29.41	2.528	342.182
Constant	− 2.538	1.057	5.769	1	0.016	0.079		

### Results of EEG data analysis

**Fig. 4:**
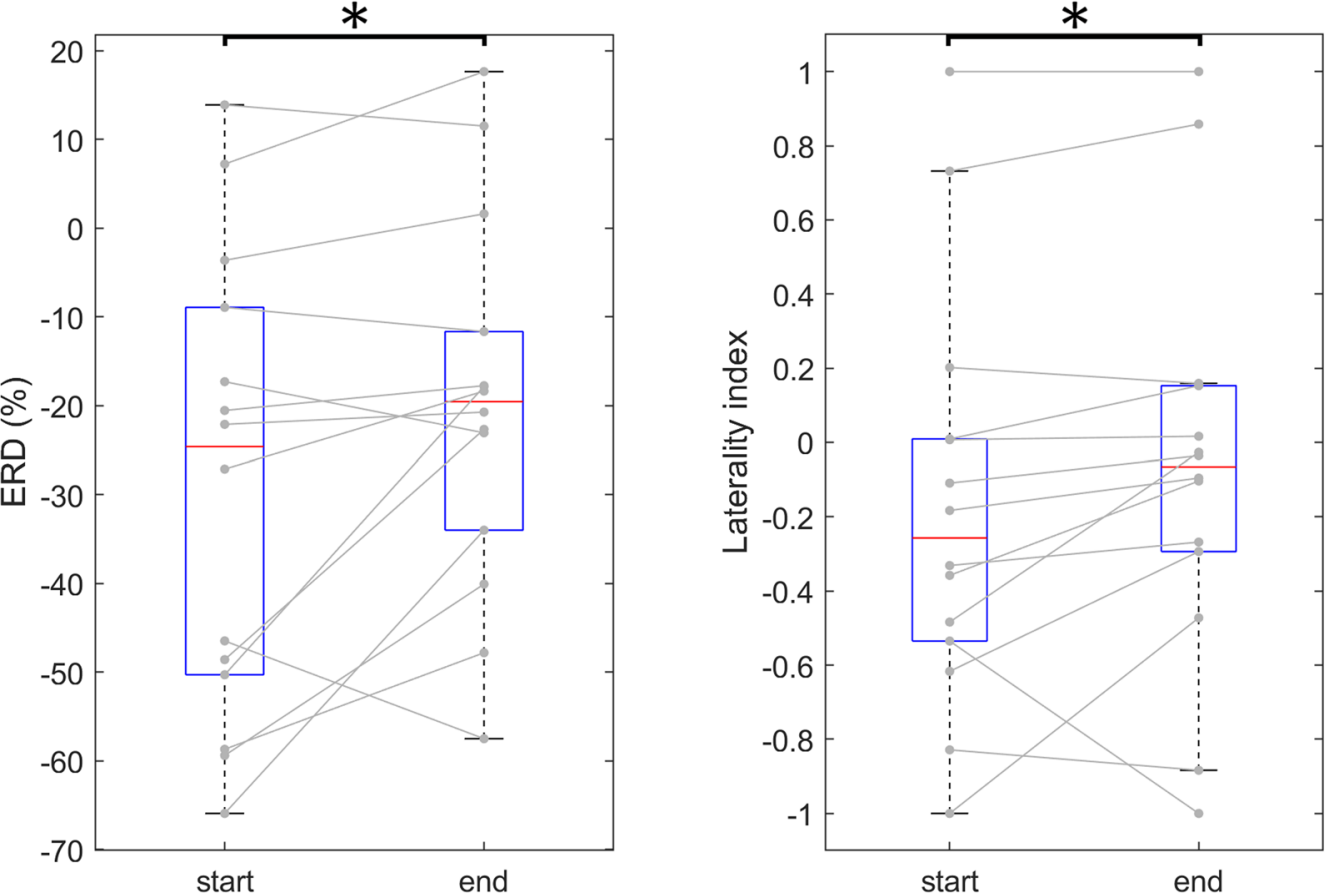
Boxplots show the EEG performance indices for each patient are depicted, namely: the left plot shows broadband (8–30 Hz) ERD for the affected hemisphere during less affected hand imagery for the values reflecting the start and end of therapy, and the right plot broadband LI during less affected hand imagery. Gray lines connect the values of the respective index computed at the start and the end of the BCI treatment for each patient. The asterisk (*) symbol is used to indicate statistically significant differences (p < 0.05) between paired values, representing the start and end of the therapy

EEG data from 15 patients in the BCI group were included in the analysis. Data from one patient was corrupted and thus excluded, leaving 14. The results revealed statistically significant differences for one ERD parameter and one LI parameter, where negative LI values reflect ipsilesional while positive LI values reflect contralesional activation. Firstly, for the less affected hand movement imagery, the mean ERD within the 8–30 Hz band in the affected hemisphere was significantly weaker at the end of therapy (− 20 ± 21%) than at the beginning (− 29 ± 26%). Secondly, for the less affected hand movement imagery, the LI within the same band was significantly higher (median {Q1, Q3}) for values reflecting the end of therapy (− 0.06 {− 0.29, 0.15}) compared to that obtained at the beginning (− 0.26 {− 0.53, 0.01}), Fig. 4.

## Discussion

In this study, we compared BCI training to standard training in patients with severe UL paresis after stroke. Slightly more patients in the BCI group (5/15) compared to the standard training group (5/20) achieved a clinically meaningful improvement of at least 6 points on ARAT. However, the difference was not statistically significant. MEP status was much more vital for future UL function than the type of treatment patients received.

This is the first study examining BCI training in the subacute phase where mainly patients with UL paralysis participated. This is a group for whom we lack effective treatments and where in general many will suffer from persistent impairments [37, 38]. Though the patients were relatively homogenous with regard to their severe UL impairment, we did not exclude patients with MEP− or unknown MEP status. The lack of UL recovery potential in a substantial part of our patients may have distorted the results. The use of biomarkers, especially TMS, to identify and stratify patients, is endorsed by several studies [29, 39] and recommended by the Stroke Recovery and Rehabilitation Roundtable [40]. Yet its use is not unequivocal since some patients with MEP− still improve their UL function. Hoonhorst et al. found no added value of TMS within the first 48 h but at 11 days after stroke [41]. In the study by Lundquist et al. 2 of 12 patients with MEP− achieved some dexterity [42]. Powell et al. applied neuromodulation in both patients with MEP+ and MEP− [43]. They found an improvement of 4.2 points on FM and 1.8 points on ARAT in MEP− patients. While these improvements are statistically significant, they are below the MCID for the assessment tools and do not indicate distal dexterity. An exception is the analysis by Senesh and Reinkensmeyer where some patients presumably without CST integrity responded to intensive training in the chronic phase [44]. Still, they did not reach advanced levels of fine motor skills. Classic experiments with non-human primates with lesions of the hand representation in the primary motor area showed that the animals regained control over arm movements, though slower, but not over selective finger movements [45]. Though alternative pathways, such as the rubrospinal tract, reticulospinal, and ipsilesional connections have been regarded as a potential means of compensating for CST damage [46, 47], CST integrity still seems to be the most decisive factor in regaining fine motor skills [40, 48]. Nevertheless, we cannot rule out that some people may be better capable of compensating for lost CST integrity, at least to some degree [44].

In our study, most patients (15/16) with MEP− did not reach any dexterity in the affected UL. This seems to be in contrast to the results by Pichiorri et al. [22], the only other study with subacute patients. In this study, 14 patients without detectable MEPs participated and many of those improved. However, it has to be taken into account that this improvement was likely limited to proximal, i.e., shoulder and upper arm function, and did not include finger movements [49]. In general, their sample included patients with better UL motor function, with a mean FMA score of 23.4 ± 17.3 in the BCI group and 24.2 ± 18.2 in control, and less overall stroke severity, a mean NIHSS score of 9 ± 2.6 in the BCI group and 8 ± 2.3 in control, than our sample with a mean FMA score of 3.4 ± 2.0 in the BCI group and 4.7 ± 5.7 in control, and a mean NIHSS score of 15.1 ± 2.7 in the BCI group and 15.9 ± 4.9 in control. Consequently, our findings corroborate the need for the stratification of patients in research and clinical applications based on the recovery potential derived from biomarkers and clinical assessments [50].

In the present study, patients received a mean of 10.6 BCI sessions. We cannot tell if more BCI sessions would have led to better results. When applied in the chronic phase, frequently a much longer treatment phase of 20–25 training sessions [20, 51, 52], or 18–30 h was offered [23]. This was not achievable in our subacute setting with a limited length of stay and patients suffering from multiple impairments which also needed to be addressed. In many cases, it was quite challenging to conduct 3–4 sessions a week. Yet, some other studies also applied a similar number of sessions. Biasucci et al. [19] conducted 10 sessions of BCI-actuated FES compared to sham FES over 5 weeks in 27 patients in the chronic phase and found significant improvement in the BCI-FES group that was retained 6–12 months after the intervention. In the above-mentioned study by Pichiorri et al. [22], 3 weekly sessions for 4 weeks were provided. Both groups improved, but there was a significantly larger improvement in the BCI group. Not only the number of BCI sessions but also the number of trials within a session could be relevant. Compared to a study by Sebastian-Ramogosa et al. [20] applying the same BCI system (RecoveriX, g.tec) in patients in the chronic phase after stroke, our patients received not only fewer sessions but also fewer runs with fewer trials within the session, only 160 as compared to 240. This was mainly caused by the lack of endurance in our subacute sample where patients suffered multiple motor and cognitive impairments, e.g., most were wheelchair users at the time of treatment. BCI training was well tolerated by these severely impaired patients when confined to 160 trials, which was still more or equal to the number of trials targeted in other studies [19, 22].

EEG analysis revealed a significant increase of LI for less affected hand movement imagery indicating an increase in contralesional activation towards the end of therapy in the broad range of movement-related frequencies. Unilateral movement imagery induces predominantly contralateral brain activation, reflected in contralateral ERD [53]. Since our results indicate a weakening of ipsilateral cortical activation for less affected hand imagery this may reflect a more natural activation pattern for less affected hand imagery towards the end of therapy, in line with both ERD and LI results. This normalization was expressed in an engagement of the lesioned hemisphere, as well as the laterality of the event-related sensorimotor oscillations during the less affected UL motor imagery over the course of BCI training. The observed changes in EEG pattern over time correspond to the statistically significant change in clinical scores on ARAT and FMA within the BCI group, implying that improvement in UL function might occur due to plastic changes within the brain. We emphasize that all patients successfully controlled the BCI system using sensorimotor alpha and beta rhythms during therapy. ERD/ERS of the sensorimotor rhythms was present in all analyzed data, confirming that the severity of impairment did not affect the presence of cortical activation in both hemispheres during UL motor imagery.

The lack of changes in the affected hemisphere for the affected hand motor imagery in our study is in contrast to findings by Pichiorri et al. who found significantly stronger contralateral ERD of the alpha and beta bands for both affected and unaffected UL imagery. However, their BCI patients had substantially higher UL function at baseline (mean FMA 23.4 ± 17.3) and experienced some recovery. We can only assume that the absence of any significant difference in event-related desynchronization (ERD) during affected hand motor imagery throughout the course of therapy may be attributed to the severe impairment and very limited recovery in our patients.

### Limitations

The main limitation of this study is the small sample size. Yet, it was conceptualized as a pilot study and these preliminary results can guide future research. A further limitation could be the relatively small number of sessions provided which was restricted by the length of stay and the patients’ general condition. Most UL recovery occurs within the first few months of stroke and preferably, the TMS examination should have been conducted at a fixed point in time within a few weeks after stroke. However, this was not possible because patients were admitted to rehabilitation and/or were medically stable enough to comply with testing and treatment procedures at various points in time. Still, as can be seen from Table 1, all included patients were in the subacute phase after stroke. The odds ratio of 29.4 for regaining motor function for the patients with MEP+ is quite large, however, it is uncertain in light of the small sample size and the large CI (2.528–342.182).

The patients were heterogeneous with regard to the type of stroke and stroke localization. While this heterogeneity can make it more challenging to draw strong conclusions, it does reflect clinical reality. We tried to balance the amount of UL training in both groups, but we could not objectively quantify it. Moreover, we did not register the amount of UL training received between discharge and the 3-month assessment. Furthermore, a selected group of patients was included, both with regard to UL impairment, contributing to the low inclusion rate, and the severity of their stroke in general. Thus, we cannot rule out that eventual additional rehabilitation and further spontaneous recovery could have influenced the outcome. However, there is no indication that there could have been a systematic difference between patients in the BCI and control groups. Therapists in municipality rehabilitation services were not aware of any study participation and the amount of spontaneous recovery that can realistically be expected 2 months after stroke is limited. Consequently, the results cannot be generalized to people with less impairment or at a later phase after stroke.

## Conclusions

We did not find a difference in UL function improvement between patients receiving BCI training and those receiving standard UL rehabilitation only. However, a substantial part of the patients included suffered from a loss of CST-integrity, which likely limited their UL recovery potential independent of the type of training. Consequently, we can neither refute nor confirm that BCI training could be effective for patients with preserved CST-integrity. Larger studies recruiting only patients with some preserved CST-integrity should be attempted. Moreover, the intensity of the BCI training provided in the present study was low. Ideally, BCI should be continued ambulatory or at home after discharge to achieve sufficient intensity. Evidence from this study may be important for further determination of the type, onset, intensity, and dose of BCI therapy in severely impaired stroke patients in the subacute phase.

## Data Availability

Data are available from the corresponding author upon request.
